# Are COPD self-management mobile applications effective? A systematic review and meta-analysis

**DOI:** 10.1038/s41533-020-0167-1

**Published:** 2020-04-01

**Authors:** G. Shaw, M. E. Whelan, L. C. Armitage, N. Roberts, A. J. Farmer

**Affiliations:** 1 Exeter College, University of Oxford, Oxford, UK; 2 Nuffield Department of Primary Care Health Sciences, University of Oxford, Oxford, UK; 3 Bodleian Health Care Libraries, University of Oxford, Oxford, UK

**Keywords:** Quality of life, Chronic obstructive pulmonary disease, Respiratory signs and symptoms, Quality of life, Chronic obstructive pulmonary disease

## Abstract

The burden of chronic obstructive pulmonary disease (COPD) to patients and health services is steadily increasing. Self-management supported by mobile device applications could improve outcomes for people with COPD. Our aim was to synthesize evidence on the effectiveness of mobile health applications compared with usual care. A systematic review was conducted to identify randomized controlled trials. Outcomes of interest included exacerbations, physical function, and Quality of Life (QoL). Where possible, outcome data were pooled for meta-analyses. Of 1709 citations returned, 13 were eligible trials. Number of exacerbations, quality of life, physical function, dyspnea, physical activity, and self-efficacy were reported. Evidence for effectiveness was inconsistent between studies, and the pooled effect size for physical function and QoL was not significant. There was notable variation in outcome measures used across trials. Developing a standardized outcome-reporting framework for digital health interventions in COPD self-management may help standardize future research.

## Introduction

Chronic obstructive pulmonary disease (COPD) affects the functional capacity of the lungs, characterized by airflow limitation and is commonly progressive^1^. One in 20 adults aged over 40 years old in the United Kingdom have diagnosed COPD and it is projected to be the fourth leading cause of global mortality by 2030^2^. Despite the preventable and treatable nature of the condition^3^, it poses a high financial burden to the healthcare systems globally. In England, the annual direct healthcare costs of COPD were estimated to be £1.5 billion in 2011, with severe exacerbations costing £3726 per event^4^. There are also substantial indirect and intangible costs associated with COPD, which are much harder to quantify, but include time lost from work, impact on family, and additional social and care costs^5^.

Acute exacerbations of COPD are defined as acute events leading to the worsening of respiratory condition beyond normal daily variation^3^. Increased frequency of exacerbations and ongoing, progressive development of the condition itself can significantly impact QoL and increase the risk of mortality^6^. Initial studies incorporating technology into self-management interventions for COPD patients combined phone calls with weekly visits from health professionals, and indicated that this strategy could result in fewer exacerbation-related hospital attendances^7^. Increasing attention to the potential for self-management has highlighted the role of digital health technologies. The capabilities of mobile device technologies have substantially increased, and applications can facilitate access to and awareness of self-management strategies for patients living with long-term conditions such as COPD.

Studies exploring patient experience and acceptability of apps have shown promise^8^, suggesting that such technology may be able to complement current clinical care. However, the evidence base to support this approach is currently unclear. Several systematic reviews have been conducted exploring applications to support self-management of COPD, but questions remain regarding their potential to improve clinical and nonclinical outcomes. Meta-analyses to date have pooled trials investigating hospital admissions^9^, physical activity^10^, physical function^10^, dyspnea^10^, and exacerbations^11^. However, reviews to date have used varying eligibility criteria for inclusion, excluding tablet computers^11^, excluding trials with any healthcare professional input^12^, excluding trials shorter than 1 month in duration^9^, or only including trials reporting hospitalization or exacerbation events^9,11^. With technologies rapidly evolving, it is also important to identify the effective and less effective components of current interventions to help inform future interventions, so this review will provide a detailed description of each intervention. The aim of this systematic review was to build on existing reviews by synthesizing and appraising evidence on the effectiveness of mobile applications (encompassing smartphones, tablet computers, and accompanying devices such as wearable sensors) in people with COPD.

## Results

### Study selection

**Fig. 1:**
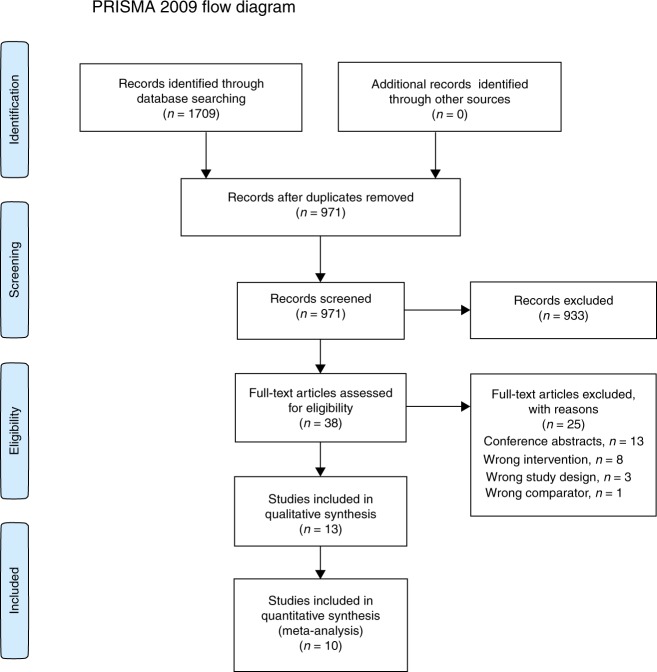
PRISMA flowchart. The PRISMA flowchart reporting the number of papers identified, screened, and excluded.

The initial search identified 1709 citations; 738 duplicates were removed. After screening titles and abstracts, 933 papers were excluded. Thirty-eight trials were assessed using full texts and 11 were deemed eligible for inclusion. After screening reference lists of the included trials, two additional trials were identified, resulting in a total of 13 trials for inclusion (Fig. 1).

### Study characteristics

Study characteristics are reported in Table 1. All 13 trials^13–24^ were published after 2008, with most (12 of 13) published since 2011. Trials were conducted in a number of countries and settings; however, most were in the Netherlands^17–20^ or the United Kingdom^13,16,22,23^. Five trials^14,17,18,23,25^ included fewer than 50 participants and the largest number of participants was 343^21^. Across all 13 trials, the total number of participants was 1447. Participants were generally aged ≥60 years, and the proportion of males and females was similar within trials. One study^25^ included male participants only and another^14^ only included one female participant. Baseline measures of lung function were identified in nine trials^13–18,20,21,25^. Study duration varied from 2 weeks^23^ to 12 months^15,16,20,22^.

**Table 1:** An overview of study characteristics of the included 13 trials, reported as mean (SD) or median (lower quartile–upper quartile), unless otherwise stated.

Author (year)	Setting	Sample size		Age		Sex (M %)		Lung function (using FEV_1_% predicted)		Primary outcome	Other outcomes	Duration of intervention	Duration of study
		Intervention	Control	Intervention	Control	Intervention	Control	Intervention	Control				
Liu (2008)	–	24	24	71.4 (1.7)	72.8 (1.3)	100	100	45.2 (3.2)	46.0 (2.8)	ISWT	Exacerbation, QoL	9 months	9 months
Halpin (2011)	Primary care	40	39	68.5 (1.5)	70.2 (1.6)	74.4	73.7	48.0 (4.0)	53.0 (3.0)	Exacerbations	Application ability to predict increases in exacerbation, changes in health status	4 months	4 months
Chau (2012)	Outpatient clinic	22	18	73.5 (6.1)	72.2 (6.1)	95.5	100	38.0 (12.9)	37.7 (16.52)	–	User satisfaction, QoL, and hospital admissions	2 months	2 months
Nguyen (2013)	Community	43	41	68.5 (11.0)	69.3 (8.0)	58.1	58.5	53.3 (20.4)	49.4 (19.8)	Dyspnea	6MWT, QoL (CRQ)	12 months	12 months
Pinnock (2013)	Primary care and community	128	128	69.4 (8.8)	68.4 (8.4)	41.4	49.2	44.0 (18.8)	40.0 (17.0)	Time to the first hospital admission due to exacerbation	Admissions, exacerbations, self-Efficacy, QoL, anxiety, and depression	12 months	12 months
Tabak (2014-A)	Primary and secondary care	12	12	64.1 (9.0)	62.8 (7.4)	50	50	50 (33.3–61.5)	36 (26–53.5)	Use of application and satisfaction	6MWT, dyspnea, fatigue, QoL, No. of hospitalizations, and activity level	9 months	9 months
Tabak (2014-B)	Secondary care	14	16	65.2 (9.0)	67.9 (5.7)	57.1	68.8	48.7 (16.7)	56.4 (10.6)	Activity level	Dyspnea, fatigue, and QoL	1 month	1 month
van der Weegen (2015)	Primary care	65	68	57.5 (7.0)	59.2 (7.5)	47.7	45.6	–	–	Activity level	QoL, general and exercise self-efficacy	4–6 months	9 months
Vorrink (2016)	Physiotherapist	84	73	62.0 (9.0)	63.0 (8.0)	50	49.3	59.0 (20.0)	53.0 (15.0)	Activity level	6MWT, QoL, and BMI	6 months	12 months
Demeyer (2017)	Community	171	172	66.0 (8.0)	67.0 (8.0)	65	63	55.0 (20.0)	57.0 (21.0)	Activity level	QoL and 6MWT	12 weeks	12 weeks
Farmer (2017)	Primary and secondary care, community	110	56	69.8 (9.1)	69.8 (10.6)	61.8	60.7	–	–	QoL	Hospital admissions, exacerbations	12 months	12 months
Orme (2018)	Secondary care	12	11	–	–	–	–	–	–	Feasibility and acceptability	Dyspnea, fatigue, anxiety, depression, QoL, and self-efficacy	2 weeks	2 weeks
Wang (2018)	Secondary care	32	32	66.4 (6.2)	67.1 (6.2)	65.6	71.9	–	–	Self-management	Dyspnea	3 months	3 months

A description of the interventions is outlined in Supplementary Table 1. Eight of the interventions^13,15,17,19,21,23–25^ were smartphone-based, using custom applications whereby participants entered COPD symptom data and received custom or automated feedback based on their responses. Healthcare professional involvement through active monitoring of entered data, clinical advice, or intervention on deteriorating observations was noted in seven trials^14,16,18–20,22,24^. Eleven trials^13–15,17–21,23–25^ delivered the intervention through a smartphone and two^16,22^ utilized a mobile tablet device. Five trials^14,18,21–23^ provided participants with a monitoring device such as a pulse oximeter and a pedometer, which linked to the applications to provide additional data.

### Risk of bias within studies

**Fig. 2:**
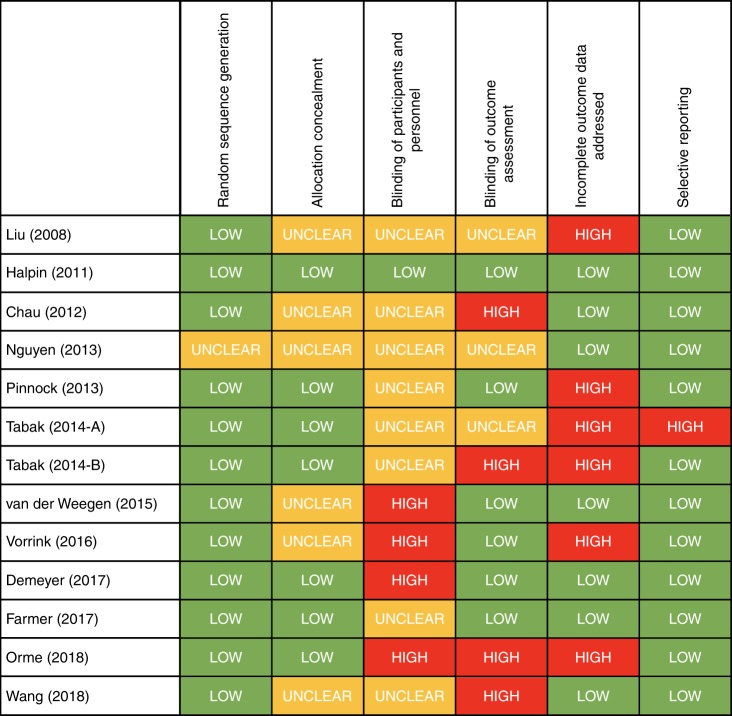
Risk of bias assessment. An outline of the bias assessment findings for the 13 included trials.

An overview of the results for the bias assessment is presented in Fig. 2. Random sequence generation was clearly carried out in 12 trials, with one trial unclear on random sequence generation^15^. Six trials^14,15,19,20,24,25^ were unclear on concealment of allocation. Risk of selective reporting was considered low in 12 trials with the remaining trial^18^ classified as having a high risk of bias. Regarding blinding of participants to intervention, four trials^19–21,23^ were considered at high risk of bias, eight trials^14–18,22,24,25^ did not provide sufficient information for assessment about the degree of participant blinding, and the remaining trial^13^ was considered at low risk. Halpin et al. (2011) was judged to be at low risk because both control and intervention participants had access to a smartphone application, with only the intervention group receiving alerts, and participants were not informed of their allocation^13^. Similarly, four trials^14,18,23,24^ were considered at high risk of bias for the blinding of outcome assessments, three trials^15,18,25^ were unclear, and the remaining six trials^13,16,19–23^ at low risk of bias.

### Primary outcome

Five trials^13,14,16,22,25^ reported the frequency of COPD exacerbations that led to clinical intervention (hospitalization or managed in the community). However, only one of these trials^14^ reported pre-intervention and post-intervention exacerbation data. One trial^16^ presented patient self-reported exacerbations but only post-intervention data. A summary of the main findings of the included trials can be seen in Table 2.

**Table 2:** A summary of the main findings for exacerbations.

Author (year) sample size	Type of exacerbation reported	Group allocation	
		Intervention	Control
Liu (2008) Intervention *N* = 24, Control *N* = 24	Managed in the community, *N*; *p* = NS	F: *N* = 4	F: *N* = 26
	Leading to hospitalization, *N*; *p* = NS	F: *N* = 2	F: *N* = 22
Halpin (2011) Intervention *N* = 40, Control *N* = 39	Clinical exacerbation frequency, mean (SD); *p* = NS	F: 0.95 (1.71)	F: 1.17 (1.81)
Chau (2012) Intervention *N* = 22, Control *N* = 18	Managed in the community, *N*; *p* = NS	F: *N* = 7	F: *N* = 3
	Leading to hospitalization, mean (SD) or *N*; *p* = NS	B: 2.41 (1.57) F: *N* = 7	B: 2.89 (2.32) F: *N* = 3
Pinnock (2013) Intervention *N* = 8, Control *N* = 8	Leading to hospitalization, mean (SD); *p* = NS	F: 1.5 (2.3)	F: 1.3 (1.8)
	Managed in the community, mean (SD); *p* = NS	F: 15 (12.7)	F: 12.8 (11.8)
Farmer (2017) Intervention *N* = 110, Control *N* = 56	Unspecified, median (IQR); *p* = NS	F: 1 (0–2)	F: 1 (0–3)

*B* baseline, *F* follow-up, *IQR* interquartile range, *N* number of participants, *NS* nonsignificant, *SD* standard deviation.

### Other outcomes

#### Physical function

**Fig. 3:**
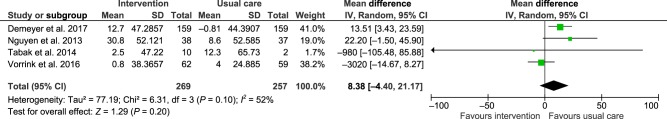
Physical function forest plot. Forest plot of the effect of mobile device applications on physical function.

Physical function was reported in five trials (Table 3)^15,18,20,21,25^. One trial^25^ recorded the incremental shuttle-walking test and showed the results that were neither statistically significant nor indicated a clinically important difference between intervention and control groups. The other trials^15,18,20,21^ used the 6-minute walk test. Only one trial^21^ recorded a significant difference between the groups in the post-intervention period. No difference between intervention and usual care was found for the 6-minute walk test (mean difference, 8.38 m, 95% CI, −4.40 to 21.17, *p* = 0.20; Fig. 3). The *I*^2^ estimate was 52% that represents moderate-to-substantial heterogeneity.

**Table 3:** A summary of the main findings for physical function.

Author (year) sample size	Type of physical function assessment reported	Group allocation	
		Intervention	Control
Liu (2008) Intervention *N* = 24, Control *N* = 24	ISWT (m), mean (SD); *p* = NS	B: 255.8 (200.9) F: 306.7 (103.9)	B: 262.9 (88.8) F: 237.8 (60.7)
Nguyen (2013) Intervention *N* = 43, Control *N* = 41	6MWT (m), mean (SD); *p* = NS	B: 400.5 (100.0) F: 431.3 (124.4)	B: 398.0 (99.7) F: 406.6 (125.0)
Tabak (2014-A) Intervention *N* = 12, Control *N* = 12	6MWT (m), mean (SD); *p* = NS	B: 409.5 (102.2) F: 412 (134.1)	B: 300.1 (116.4) F: 312.4 (152.4)
Vorrink (2016) Intervention *N* = 84, Control *N* = 73	6MWT (m), mean (SD) or median (IQR); *p* = NS	B: 456 (128.3) C: 0.8 (−8.8 to 10.3)	B: 461 (73.3) C: 4 (−2.4 to 10.3)
Demeyer (2017) Intervention *N* = 171, Control *N* = 172	6MWT (m), mean (SD); *p* = 0.009	B: 444 (106) F: 457 (108)	B: 450 (106) F: 449 (118)

*6MWT* 6-minute walk test, *B* baseline, *C* change, *F* follow-up, *IQR* interquartile range, *m* meters, *NS* nonsignificant, *SD* standard deviation.

#### Quality of life (QoL)

**Fig. 4:**
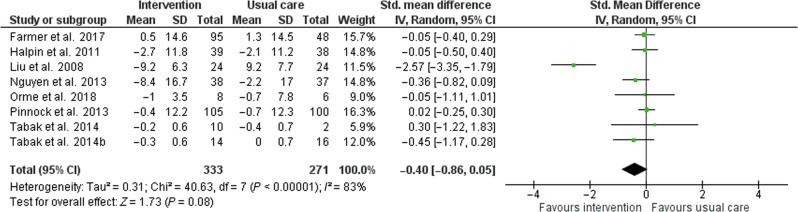
QoL forest plot. Forest plot of the effect of mobile device applications on quality of life.

Twelve of the 13 trials reported QoL; two of these trials^15,21^ reported two different quality-of-life measures. Across all 12 trials, 14 quality-of-life measures were reported (Table 4). Only one trial^25^ reported the SF-12 measure, reporting a significant difference between intervention and control post-intervention. Two trials^15,19^ used the SF-36 measure, but these did not identify statistically significant differences. One trial^21^ reported the individual mental, functional, and symptom domains of the Chronic COPD Questionnaire. There was a significant difference between the intervention and control groups in the Functional CCQ measure post intervention but not in other domains. Two trials^17,18^ recorded the total CCQ score, but the results were not significant. The Chronic Respiratory Disease Questionnaire was reported in full by one trial^15^, and partially by two trials^14,20^ (only reporting the emotion and mastery domains). These three trials reported non-significant results for these domains. Three trials^13,16,22^ reported the St. George’s Respiratory Questionnaire and two trials^21,23^ reported the COPD Assessment Test measure of QoL, but none of them showed significant differences between intervention and control groups. The 12 trials reporting QoL were assessed for inclusion for the meta-analysis, but trials that did not report a total or summative score were excluded, resulting in a total of eight eligible trials (Fig. 4). The trial by Nguyen et al. (2013) reported two total scores reflecting QoL (Chronic Respiratory Disease Questionnaire and SF-36); the disease-specific scale (Chronic Respiratory Disease Questionnaire) was included in the meta-analysis. No difference in QoL was found between mobile device application intervention and usual care (standardized mean difference, −0.4 points; 95% CI, −0.86 to 0.05, *p* = 0.08). The *I*^2^ estimate was 83% that represents considerable heterogeneity. The minimal clinically important differences for the back-translated standardized mean differences are presented in Supplementary Table 2.

**Table 4:** A summary of the main findings for quality of life.

Author (year) sample size	Form of QoL assessment reported	Group allocation	
		Intervention	Control
Liu (2008) Intervention *N* = 24, Control *N* = 24	SF-12 PCS, mean (SD); *p* < 0.001	B: 38.7 (8.82) F: 47.9 (7.35)	B: 40.1 (6.37) F: 30.9 (10.78)
Halpin (2011) Intervention *N* = 40, Control *N* = 39	SGRQ, mean (SD); *p* = NS	B: 52.4 (16.44) F: 49.7 (15.18)	B: 53.6 (14.99) F: 51.5 (14.99)
Chau (2012) Intervention *N* = 22, Control *N* = 18	CRQ (Emotion), mean (SD); *p* = NS	B: 4.84 (1.47) F: 4.92 (1.40)	B: 5.24 (1.42) F: 5.61 (1.17)
	CRQ (Mastery), mean (SD); *p* = NS	B: 4.60 (1.43) F: 4.61 (1.62)	B: 4.94 (1.16) F: 4.88 (1.27)
Nguyen (2013) Intervention *N* = 43, Control *N* = 41	CRQ (Total), mean (SD); *p* = NS	B: 96.4 (19.91) F: 104.8 (23.92)	B: 96.2 (19.76) F: 98.4 (24.34)
Pinnock (2013) Intervention *N* = 8, Control *N* = 8	SGRQ, mean (SD); *p* = NS	B: 68.6 (16.6) F: 68.2 (16.3)	B: 68.0 (15.2) F: 67.3 (17.3)
Tabak (2014-A) Intervention *N* = 12, Control *N* = 12	CCQ (Total), mean (SD); *p* = NS	B: 2.0 (0.90) F: 1.8 (0.83)	B: 2.7 (0.94) F: 2.3 (0.90)
Tabak (2014-B) Intervention *N* = 14, Control *N* = 16	CCQ (Total), mean (SD); *p* = NS	B: 2.0 (0.8) F: 1.7 (0.5)	B: 1.8 (1.0) F: 1.8 (0.6)
van der Weegen (2015) Intervention *N* = 65, Control *N* = 68	SF-36 (Physical), mean (SD); *p* = NS	B: 42.5 (11.1) F: 44.1 (9.5)	B: 45.8 (9.4) F: 45.8 (9.5)
	SF-36 (Mental), mean (SD); *p* = NS	B: 48.2 (10.3) F: 48.3 (11.7)	B: 50.1 (9.5) F: 50.3 (8.3)
Vorrink (2016) Intervention *N* = 84, Control *N* = 73	CRQ (Emotion), mean (SD) or median (IQR); *p* = NS	B: 5.0 (1.1) C: 0.09 (−0.07 to 0.24)	B: 4.8 (1.2) C: 0.19 (−0.31 to 0.11)
	CRQ (Mastery), mean (SD) or median (IQR); *p* = NS	B: 5.4 (1.1) C: −0.1 (−0.31 to 0.11)	B: 5.3 (1.1) C: −0.23 (−0.39 to −0.06)
Demeyer (2017) Intervention *N* = 171, Control *N* = 172	CCQ (Mental), median (IQR); *p* = NS	B: 1 (0–2.5) F: 1 (0–2.5)	B: 1 (0–2) F: 1 (0–2)
	CCQ (Functional), median (IQR); *p* = 0.026	B: 1.5 (1–2.75) F: 1.5 (1–2.75)	B: 1.5 (0.75– 2.75) F: 1.75 (0.75– 2.75)
	CCQ (Symptoms), median (IQR); *p* = NS	B: 1.75 (1.25– 2.5) F: 1.75 (1.25– 2.5)	B: 1.75 (1.5–2.75) F: 2 (1.25– 2.75)
Farmer (2017) Intervention *N* = 110, Control *N* = 56	SGRQ, mean (SD); *p* = NS	B: 56.4 (19.7) F: 56.9 (19.5)	B: 55.5 (16.2) F: 56.8 (20.9)
Orme (2018) Intervention *N* = 12, Control *N* = 11	CAT, mean (SD); *p* = NS	B: 22.6 (4.4) F: 21.6 (5)	B: 24.5 (9.7) F: 23.8 (11.1)

*B* baseline, *C* change, *CCQ* Clinical COPD Questionnaire, *CRQ* Chronic Respiratory Disease Questionnaire, *F* follow-up, *SGRQ* St. George's Respiratory Questionnaire, *SF* short form, *CAT* COPD Assessment Test, *IQR* interquartile range, *SD* standard deviation, *NS* nonsignificant.

#### Dyspnea

Five trials^14,15,17,20,24^ reported data relating to dyspnea (Supplementary Table 3). Three trials^14,15,20^ used the dyspnea component of the Chronic Respiratory Disease Questionnaire measure, while the other two trials^17,24^ used the modified Medical Research Council dyspnea scale. Only one trial^24^ reported a statistically significant difference between groups.

#### Fatigue

Five trials^14,17,18,20,23^ reported data concerning fatigue (Supplementary Table 4). Two trials^14,20^ reported the fatigue component of the CRQ, two trials^17,18^ reported the Multidimensional Fatigue Inventory, and one trial^23^ used the Functional Assessment of Chronic Illness Therapy measure. None of these trials reported significant improvements in the intervention arm compared with control.

#### Physical activity

Five trials^17–21^ reported device-based levels of physical activity (Supplementary Table 5). Four trials recorded physical activity using accelerometers, while the remaining trial used pedometers. Only one trial^19^ reported a statistically significant difference in physical activity outcomes between groups in the post-intervention period. Two of these five trials also provided self-reported levels of physical activity, using the Moderate Physical Activity questionnaire^21^ and the Baecke Physical Activity Questionnaire^18^. Both trials reported non-significant changes from baseline.

#### Self-efficacy

Four trials^15,16,19,23^ reported patient self-efficacy (Supplementary Table 6). The employed measures focused on dyspnea^15^, falls^23^, exercise^19^, and self-efficacy more generally^16,19^. No trials recorded statistically significant findings.

#### Anxiety and depression

Two trials^16,23^ reported anxiety and depression, using the Hospital Anxiety and Depression Scale (HADS), and no statistically significant differences were observed.

## Discussion

This systematic review provided a comprehensive description and summarized the findings of mobile device application interventions for COPD self-management. The interventions identified were heterogeneous in nature, including the components (such as the inclusion of periphery devices), the degree and frequency of involvement of healthcare professionals, and frequency of participant-performed measurements and data entry. It remains unclear whether mobile device applications are more effective at preventing exacerbations when compared with usual care.

As only published trials were eligible for inclusion, there is potential for publication bias within the review. Also, the risk assessment bias tool was challenging to implement because blinding of participants in digital health interventions where the comparator is usual care may not be feasible to implement. In addition, our ability to pool further outcome measures using meta-analysis was limited, given the variety of outcome measures used across the trials. There are also limitations to interpreting summary estimates from pooled data, particularly when the design of the studies, scales used to assess effectiveness, and interventions tested are heterogeneous and use varying follow-up durations. However, the present review was prospectively registered on a database of systematic reviews and included trials published in any language in several databases from inception. A sensitive search strategy was developed, and screening of citations was performed independently, minimizing the risk of bias at review level. The review was inclusive of a broad range of outcome measures, contributing to its comprehensive nature.

Although exacerbations can negatively impact QoL^26^ and increase mortality^27^, only five of the included trials reported exacerbations. Only one of these trials reported pre-intervention and post-intervention exacerbation frequency^14^, and exacerbations were reported using a wide range of metrics, including those exacerbations managed in the community and leading to hospitalization. An 80% reduction in likelihood of having an exacerbation has been demonstrated previously in a meta-analysis comparing a smartphone intervention with usual care^11^. However, the meta-analysis showed moderate heterogeneity in this healthcare professional contact, in part possible because of the small sample size of the three trials pooled. It is unclear if reporting the number of contacts with healthcare professionals is a suitable outcome measure to represent COPD exacerbations; digital interventions can offer an alternate means of contacting a healthcare professional, impacting the accuracy of assessing exacerbation frequency in this way. With prevention and management of exacerbations being a key feature of COPD care, and an increasing interest in predicting the onset of exacerbations^28–30^, future trials are recommended to consider this when reporting exacerbations to more accurately quantify the impact of digital interventions on this important clinical outcome.

The trials identified in this systematic review do not yet provide strong evidence for implementing mobile digital health interventions for COPD. Only four trials reported clinical differences between the intervention and control groups, and these differences were in a range of outcomes, including physical function, QoL, physical activity, and dypsnea^19,21,25,31^. This apparent lack of impact may be from the small size of the studies, with 8 of the 13 trials reporting a sample size of fewer than 100 participants^13–15,17,18,23,25,31^. In addition, the extent to which the measures used in these studies were sensitive to change is unclear.

Hanlon et al. conducted a metareview of telehealth trials across multiple health conditions, including COPD, diabetes, cancer, and heart failure^32^. Their findings suggest that the evidence base is more developed in diabetes and heart failure and more intensive and multifaceted interventions associated with greater improvements in asthma, diabetes, and heart failure. Building on published reviews focused on COPD, our findings also report on QoL, self-efficacy, fatigue, anxiety, and depression, as well as exacerbations, physical function, and physical activity. In addition, we provide an in-depth description of the interventions within the included trials.

The results from our pooled data meta-analysis do not identify a statistically significant effect on measures of physical function or QoL. Previous meta-analyses have identified no differences in physical function (using the 6-minute walk test)^10^, dyspnea^10^, and average days of hospitalization^9^, but have noted that the intervention arm was favored for physical activity^10^ and a lower risk of hospital admissions^9^.

Looking beyond the effectiveness of the intervention for clinical outcomes, it is possible that there are efficiency and organizational benefits of digital and telehealth care compared with more traditional models of care. None of the studies included in this review reported service outcomes.

The trial interventions identified in our review focused on varying components of COPD self-management, including monitoring symptoms, encouraging lifestyle changes (such as increases in physical activity or exercise), and hosting educational material concerning COPD. Some of the trials explored ease of use, feasibility, and accessibility of the technologies. Aligning with this heterogeneity is the variety of outcome measures used to assess the effectiveness of the intervention. This review highlights the number of outcome measures used and variation in which the tool was used for data collection between studies.

Our findings and the challenges encountered in synthesizing the evidence from these trials highlight the importance of developing a minimum and standardized set of clinically important core-outcome measures to allow comparison of trials involving people with COPD. This would be in line with minimum reporting guidelines for other areas of clinical speciality, including rheumatology^33^. In practice, the use of mobile device applications to support self-management may have some negative effects. For example, a patient might be falsely reassured if they feel their data were being monitored by a healthcare professional. On the other hand, the data can supplement routine care with information about variation in symptoms and clinical markers of the condition. From a policy perspective, the economic cost of telehealth for chronic disease is high (£92,000/QALY), which restricts its implementation in the majority of healthcare settings^34^.

In conclusion, this systematic review demonstrates that there are a number of trials being conducted in this area of COPD. However, there is insufficient evidence to date to suggest that mobile device applications are effective for the self-management of COPD over usual care. This may in part be due to a limited ability for data to be pooled, owing to marked variation in methodology and reporting of outcome measures. Future efforts to standardize the outcomes used in this area of research are encouraged to increase the comparability of future trials.

## Methods

### Registration

The review was registered on the International Prospective Register of Systematic Reviews (PROSPERO reference number: CRD42019124232).

### Eligibility criteria

Randomized controlled trials of adults with a clinical diagnosis of COPD were included where the intervention group received a mobile device application to support their COPD self-management. A mobile device application was defined as a contained program that served a specific function relating to COPD and personal health on a portable, electronic device (including smartphones and tablet computers). This definition is in line with previous systematic reviews on the topic^11,12^. For the purpose of inclusion, self-management was defined as patient management of their personal symptoms and medication regimes related to the condition, as well as coping with the emotional and lifestyle impacts of the condition^35,36^. Studies were eligible where the comparator group received usual care only. Outcomes included but were not restricted to exacerbations, QoL, physical function, physical activity, and dyspnea.

### Information sources and search

Medline, EMBASE, Cochrane Library, CINAHL, and the Science Citation Index were searched from inception to 12th April 2019 following the methods recommended by the Preferred Reporting Items for Systematic Reviews and Meta-Analyses guidelines^37^. Full search strategies are included in Supplementary Methods. The search algorithm focused on keywords relating to ‘COPD’, ‘mobile phone application’, and ‘self-management’ and included interventions with or without healthcare professional input.

### Study selection

The resulting citations were imported into the web-based Covidence systematic review software (Veritas Health Innovation, Melbourne, Australia). Screening of titles and abstracts was completed by two authors independently (G.S. and M.W.). In the event of disagreement, two further reviewers (L.A. and A.F.) decided their eligibility. Subsequently, full-text screening was conducted by two authors independently (G.S. and M.W.). Any disagreements were resolved following discussion with the other reviewers (L.A. and A.F.). The reference lists of the included trials were also screened to identify any additional potentially eligible trials.

### Data collection process

Extraction forms were used to capture the following data: lead author, year, country, trial setting, sample size, age, sex, lung function, primary and secondary outcomes, duration of intervention and study, as well as the main findings. Data extraction was completed independently by two authors (G.S. and M.W.), and any disagreements were resolved through discussion. When data were not directly identifiable within text or tables, authors were contacted or Microsoft Paint (Microsoft, Washington, USA) was used to extract values from graphs. The graphical summaries were captured by screenshot and copy-pasted into the software. No correction for rotation was required. Horizontal lines were inserted across from the center of the datapoints of interest to the point of intersection on the *y*-axis. The *y*-axis was segmented into smaller increments, marked by adding small lines to the axis, until a value could be extracted to 1 decimal place. The values were extracted from the original *y*-axis scale, meaning the *x* and *y* positions were not translated. Two authors (G.S. and M.W.) independently looked at the graphs to identify the value of interest. In the event any disagreements were identified, G.S. and M.W. reassessed the graphs and agreed on a value.

We subsequently replicated the data extraction using web plot digitizer software (Automeris version 3.9, https://automeris.io/WebPlotDigitizer/). The graphical summaries were captured by screenshot and saved as a PNG file before being uploaded to the web-based plot digitizer software. No correction for rotation was required. Once uploaded, two anchoring points were assigned to each axis: the highest and lowest value on the *y*-axis and baseline and follow-up for the *x-*axis. Values reflecting these anchoring points were declared. The datapoints were selected using the center of each point to 14 decimal places, and the acquired data were recorded in the form of coordinates that aligned with the scales in the original graphs.

### Risk of bias assessment

The included trials were assessed for potential bias at study level using the Cochrane risk of bias tool^38^. Two authors (G.S. and M.W.) independently completed the assessment of bias, and any disagreements were resolved through discussion with the other reviewers (L.A. and A.F.).

### Synthesis of data

The results were converted to mean (standard deviation) when possible; otherwise data were reported as median (lower to upper quartile). A pragmatic decision was made to include outcome measures reported by four or more trials in the main table and those reported less frequently in the text. Where the duration of intervention period and study duration differed, data were extracted for the end of the observation period. Outcomes were grouped together where different measures were used, for example, where different scales for QoL measurement were used. The total scores from the QoL measurement tools were extracted when these were reported; otherwise individual component scores were extracted. Similarly, exacerbations that were treated in the community were grouped, to include self-reported exacerbations (where a participant may have initiated a rescue pack), alongside exacerbations that were managed by primary care teams. Measures of physical activity were included in the summary table if these were objectively measured; self-report of physical activity was not included.

### Synthesis of results

Meta-analysis was carried out using Review Manager (Review Manager [RevMan] version 5.3, Cochrane Collaboration, Copenhagen, Denmark). A difference-in-difference random effect analysis was used to help control for differences between trials, and to limit the impact of heterogeneity. Trials were weighted by sample size, and 95% confidence intervals were reported around point estimates. Measures were selected for inclusion if they were reported by at least three trials to align with the recent Cochrane review^12^. For continuous data with consistent units of measurements (such as the 6-minute walk performance in meters), the mean difference in change between baseline and follow-up measurements was calculated. In instances where continuous data were inconsistent between trials (i.e., multiple questionnaires with varying scales used to measure QoL), the standardized mean differences between timepoints were calculated. Back-translation of the standardized mean difference for each scale was conducted to the original scale, to present a mean difference for each QoL instrument to give information of the clinical significance of this difference. Where change in standard deviation was not reported by individual trials, the standard deviation for changes from baseline was imputed by calculating a correlation coefficient from trials reporting a change in standard deviation. If the data were not reported, authors were contacted to access this information. The *I*^2^ statistic was used to estimate heterogeneity. Cochrane recommendations for interpreting the *I*^2^ statistic are as follows: 30–60% may represent moderate heterogeneity, 50–90% may represent substantial heterogeneity, and 75–100% may represent considerable heterogeneity^39^. No funnel plot was produced as it is not recommended for meta-analyses with fewer than 10 trials^40^.

### Reporting summary

Further information on research design is available in the Nature Research Reporting Summary linked to this article.

## Supplementary information

Supplementary Information

Reporting Summary

## Data Availability

All data generated or analyzed during this study are included in this published article and Supplementary Material files.

## Code availability

No custom code or mathematical algorithm were used.
