# Supplementary material for: Are COPD self-management mobile applications effective? A systematic review and meta-analysis
Source: NPJ Prim Care Respir Med. 2020 Apr 1;30:11. doi: 10.1038/s41533-020-0167-1 (PMC7113264; doi:10.1038/s41533-020-0167-1)
Supplement: Supplementary file 1 — Supplementary Information [file 41533_2020_167_MOESM1_ESM.pdf]

SUPPLEMENTARY

Search strategy for Cinahl

#	Searches	Results
S1	(MH "Pulmonary Disease, Chronic Obstructive+") OR (MH "Emphysema")	11,342
S2	TI (COPD or Chronic obstructive pulmonary disease or Chronic obstructive airway disease or Chronic obstructive lung disease or COAD or Pulmonary emphysema or Chronic bronchitis or Obstructive lung disease) OR AB (COPD or Chronic obstructive pulmonary disease or Chronic obstructive airway disease or Chronic obstructive lung disease or COAD or Pulmonary emphysema or Chronic bronchitis or Obstructive lung disease)	12,599
S3	S1 OR S2	15,479
S4	(MH "Mobile Applications") OR (MH "Computers, Hand-Held+")	5,710
S5	(MH "Cellular Phone") OR (MH "Smartphone")	1,594
S6	TI (Mobile or App or Apps or "Mobile application*" or mHealth or tablet* or smartphone* or cellphone* or "smart phone*" or "cell phone*" or "personal digital assistant*" or PDA or iPhone* or ipad* or "mobile health" or m-health) OR AB (Mobile or App or Apps or "Mobile application*" or mHealth or tablet* or smartphone* or cellphone* or "smart phone*" or "cell phone*" or "personal digital assistant*" or PDA or iPhone* or ipad* or "mobile health" or m-health) or TI (online or electronic) or AB ((online or electronic) and ("face to face" or "face 2 face" or f2f or f-2-f))	37,770
S7	S4 OR S5 OR S6	40,510
S8	(MH "Self Care")	25,489
S9	TI (Self-manag* or "Self manag*" or Self-monitor* or "Self monitor*" or "self care") OR AB (Self-manag* or "Self manag*" or Self-monitor* or "Self monitor*" or "self care") OR TI (monitor* or manage* or exacerbation* or "pulmonary rehabilitation") OR AB (monitor* or manage* or exacerbation* or "pulmonary rehabilitation")	325,636
S10	S8 OR S9	338,585
S11	S3 AND S7 AND S10	**47**

Search strategy for Cochrane

#	Searches
1	MeSH descriptor: [Pulmonary Disease, Chronic Obstructive] explode all trees
2	COPD or "Chronic obstructive pulmonary disease" or "Chronic obstructive airway disease" or "Chronic obstructive lung disease" or COAD or "Pulmonary emphysema" or "Chronic bronchitis" or "Obstructive lung disease":ti,ab,kw (Word variations have been searched)
3	#1 or #2
4	MeSH descriptor: [Mobile Applications] explode all trees
5	MeSH descriptor: [Cell Phone] explode all trees

6	MeSH descriptor: [Computers, Handheld] explode all trees
7	Mobile or App or Apps or "Mobile application*" or mHealth or tablet* or smartphone* or cellphone* or "smart phone*" or "cell phone*" or "personal digital assistant*" or PDA or iPhone* or ipad* or "mobile health" or m-health:ti,ab,kw (Word variations have been searched)
8	(online or electronic):ti
9	((online or electronic) and ("face to face" or "face 2 face" or f2f or f-2-f)):ti,ab,kw
10	#4 or #5 or #6 or #7 or #8 or #9
11	MeSH descriptor: [Self Care] explode all trees
12	Self-manag* or "Self manag*" or Self-monitor* or "Self monitor*" or "self care":ti,ab,kw or monitor* or manage* or exacerbation* or "pulmonary rehabilitation":ti,ab,kw (Word variations have been searched)
13	#11 or #12
14	#3 and #10 and #13
15	MeSH descriptor: [Telerehabilitation] explode all trees
16	#3 and #15
17	#14 or #16

Search strategy for Embase

#	**Searches**	**Results**
1	obstructive airway disease/ or chronic obstructive lung disease/ or chronic bronchitis/	120081
2	(COPD or Chronic obstructive pulmonary disease or Chronic obstructive airway disease or Chronic obstructive lung disease or COAD or Pulmonary emphysema or Chronic bronchitis or Obstructive lung disease).ti,ab.	109362
3	1 or 2	150159
4	Mobile Application/	6041
5	exp mobile phone/	18988
6	personal digital assistant/	1231
7	(Mobile or App or Apps or Mobile application? or mHealth or tablet? or smartphone? or cellphone? or smart phone? or cell phone? or personal digital assistant? or PDA or iPhone? or ipad? or mobile health or m-health).ti,ab.	226285
8	(online or electronic).ti.	55808
9	((online or electronic) and ("face to face" or "face 2 face" or f2f or f-2-f)).ti,ab.	3073
10	4 or 5 or 6 or 7 or 8 or 9	287535
11	self care/ or self monitoring/	53610
12	(Self-manag* or Self manag* or Self-monitor* or Self monitor* or self care).ti,ab.	48519
13	(monitor* or manage* or exacerbation* or pulmonary rehabilitation).ti,ab.	2408479
14	11 or 12 or 13	2438653
15	3 and 10 and 14	540

16	telerehabilitation/	391
17	3 and 16	25
18	15 or 17	**562**

Search strategy for Medline

#	Searches	Results
1	exp Pulmonary Disease, Chronic Obstructive/	48513
2	(COPD or Chronic obstructive pulmonary disease or Chronic obstructive airway disease or Chronic obstructive lung disease or COAD or Pulmonary emphysema or Chronic bronchitis or Obstructive lung disease).ti,ab.	67951
3	1 or 2	83407
4	Mobile Applications/	3091
5	exp Cell Phone/	8738
6	exp Computers, Handheld/	5092
7	(Mobile or App or Apps or Mobile application? or mHealth or tablet? or smartphone? or cellphone? or smart phone? or cell phone? or personal digital assistant? or PDA or iPhone? or ipad? or mobile health or m-health).ti,ab.	159405
8	(online or electronic).ti.	57554
9	((online or electronic) and ("face to face" or "face 2 face" or f2f or f-2-f)).ti,ab.	2220
10	4 or 5 or 6 or 7 or 8 or 9	221181
11	Self Care/	30437
12	(Self-manag* or Self manag* or Self-monitor* or Self monitor* or self care).ti,ab.	34642
13	(monitor* or manage* or exacerbation* or pulmonary rehabilitation).ti,ab.	1771007
14	11 or 12 or 13	1794517
15	3 and 10 and 14	261
16	Telerehabilitation/	172
17	3 and 16	13
18	15 or 17	**269**

Search strategy for Science Citation Index

#	Searches	Results
1	TS=(COPD or "Chronic obstructive pulmonary disease" or "Chronic obstructive airway disease" or "Chronic obstructive lung disease" or COAD or "Pulmonary emphysema" or "Chronic bronchitis" or "Obstructive lung disease")	72,566
2	TS=(Mobile or App or Apps or "Mobile application*" or mHealth or tablet* or smartphone* or cellphone* or "smart phone*" or "cell phone*" or "personal digital assistant*" or PDA or iPhone* or ipad* or "mobile health" or m-health) or TI=(online or electronic) or TS=((online or electronic) and ("face to face" or "face 2 face" or f2f or	730,527

f-2-f))

3	TS=(Self-manag* or "Self manag*" or Self-monitor* or "Self monitor*" or "self care") OR TS=(monitor* or manage* or exacerbation* or "pulmonary rehabilitation")	3,225,087
4	#3 AND #2 AND #1	**316**

Supplementary Table 1

Supplementary Table 1

			Intervention			
**Author**	**Country**	**Usual care Brief description**	**Communication device(s)**	**Measurement device(s)**	**Data transmission and management**	**Training and instruction**
Liu, 2008	Taiwan	Verbally asked to take daily walking exercise at home.	Phone and web-based.	Phone recorded duration of music played, and participants answered daily symptom diaries.	Music pacing for daily endurance exercise exercise was provided to the participant through the phone, and adherence and compliance of the program was assessed on a website.	Programme booklet and DVD provided. Participants asked to take daily endurance exercise training. No telephone reinforcement provided.
Halpin, 2011	UK	Completed daily diary on a smartphone but did not receive alert calls.	Phone.	Participants answered daily symptom diaries.	Participants alerted if at higher risk of an exacebation via an automated telephone call. Automated calls were made on Tuesday evenings. Symptom diary entries were also monitored with follow up telephone and home visits scheduled.	All participants received the Met Office information pack and a telephone call to demonstrate how the system worked. Symptom diaries were delivered at 16:00 each day and participants were prompted to complete them that evening. A reminder was given if no response was received.
Chau, 2012	Hong Kong	Nothing additional to usual care.	Phone.	Pulse oximeter and respiratory rate sensor.	Data transmitted to an online network platform. Reminders for taking medication and to conduct purse-lip breathing were presented. A community nurse monitored changes in the physiological parameters and took immediate action.	Community nurses instructed participants on the use of the device for self-monitoring through demonstration and return demonstration. Participants were asked to monitor their oxygen saturation, pulse rate and respiration rate using the device three times a day on weekdays.
Nguyen, 2013	USA	Participants participated in monthly face-to-face education classes (or mailed the educational materials) that focused on health topics unrelated to lung disease. Participants also received biweekly phone calls that provided general health information.	Phone and web-based.	Participants answered symptom and exercise diaries.	Participants were encouraged to communicate their exercise goals and progress to the nurse using a web-based goal-setting tool. The nurses used this information to provide individualized feedback and reinforcement to participants regarding their use of dyspnea management strategies and exercise progress via email for the first month then biweekly for remaining period. Automated real-time e-mail alerts were sent to the nurses if eDSMP participants reported worsening of symptoms.	Training provided on the website and smartphone. Unsupervised independent exercise 4 times per week, 30 min sessions). Structured education via interactive web modules and live group chat sessions (six 1-hour sessions).
Pinnock, 2013	UK	Education on self management of exacerbations was provided, reinforced by a British Lung Foundation booklet about living with COPD. Identical clinical care (including self management advice) was delivered according to the region in which they lived.	Tablet computer.	Pulse oximeter and participants anasked to enter daily symptom diaries.	The supporting clinical team monitored incoming data online on a daily basis.	The telemonitoring equipment and secure broadband link was installed in the homes of intervention participants. The clinical team visited participants to explain how to use the technology and provide self management education.
Tabak, 2014 A	Netherlands	Nothing additional to usual care.	Phone and web-based.	Activity monitor and participants were asked to enter daily symptom diaries.	Graphs and motivational messages presented via the smartphone and web portal. Both primary and secondary care professionals could supervise the patient at a distance by checking progress with the exercise program, symptom diary responses and physical activity on the web portal. Teleconsultation module available for comments and asking questions via the web portal.	Patients received instructions from the primary care physiotherapist explaining the web portal.
Tabak, 2014 B	Netherlands	Nothing additional to usual care.	Phone and web-based.	Activity monitor and participants were asked to enter daily symptom diaries.	Activity feedback was presented via the monitor and through the web portal. Users automatically received feedback text messages, for awareness and extra motivation.	Participants asked to use the activity coach for a minimum of 4 days per week from waking to 22:00. Participants had to attend two 90-minute self-management sessions given by a nurse practitioner, to learn how to complete the daily diary, how to recognize symptoms of an impending exacerbation, and how to deal with the exacerbation.
van der Weegen, 2015	Netherlands	Nothing additional to usual care.	Phone and web-based.	Activity monitor and participants were asked to enter levels of enjoyment and exertion in performed activities.	Real-time and historical physical activity levels presented in relation to a personal goal. Activity results and answers to dialogue sessions were visible for the practice nurse on a secured web app.	Participants asked to wear the activity monitor every day. Participants received a general and a disease-specific pamphlet about physical activity and information about locally organised activities.
Vorrink, 2016	Netherlands	Nothing additional to usual care.	Phone.	Activity monitor (built in smartphone).	Physiotherapists accessed a website to monitor incoming data. Participants received real-time feedback on physical activity levels via smartphone.	Participants given instruction on the use of the smartphone and the application. The subjects in the intervention group were instructed to wear the smartphone in a pouch on their belt and use it as their usual phone.
Demeyer, 2017	Belgium, Greece, UK, Switzerland, Netherlands	Participants received a standard leaflet explaining the importance of physical activity in COPD as well as recommendations. This leaflet was discussed with all patients in a 5–10 min one-to-one discussion with the investigator.	Phone.	Activity monitor (pedometer).	Step count was presented by the pedometer and via the app. The feedback included a graphical representation of that day's performance and an educational tip. Telephone contacts were triggered in the case of non-compliance with wearing the step counter, failure to transmit data or failure to progress. Participants also received weekly group text messages.	A booklet containing home exercises was provided.
Farmer, 2017	UK	Participants were provided with all the information given to those allocated to the intervention group, but without the use of a tablet computer or the facility for daily monitoring. Participants were provided with leaflets based on those currently produced by the local community respiratory service.	Tablet computer and web-based.	Pulse oximeter and participants were asked questions about symptoms (daily) and mood (monthly).	Oxygen saturation and symptom diary scores presented via the tablet computer. Entered data was remotely monitored twice weekly. Alerts were generated in relation to the vital signs data and total symptom scores.	Participants were briefly instructed on the use of the platform by the research nurse and given a brief information booklet detailing its use.
Orme, 2018	UK	Usual care plus an educational booklet about reducing time spent sedentary was provided.	Phone.	Activity monitor.	Data relating to time spent sitting presented and vibration prompts were in place to encourage movement at patient-defined intervals of time.	Educational booklet about reducing time spent sedentary was provided. Participants were shown how to use the technology by a researcher.
Wang, 2018	China	Participants received a monthly phone call asking about health in particular cough/sputum/breathing and received a leaflet about how to inhale medicine, improve breathability and use oxygen at home.	Phone.	Participants answered daily symptom, oxygen, medication, sport and exercise diaries.	Staff could access participant entered data. If no entries were received for 3 consecutive days then participants were reminded by online chat/telephone to respond to the questions. Staff also made phone calls to participants and give participant suggestions by telephone or online chat based on data entered by participant.	No training or instruction described.

Supplementary Table 2

Quality of life scale	Back-translated mean difference	MCID	Source of MCID
SGRQ	10.71 units	4 units	Jones, 2005
CCQ	0.51 units	0.4 units	Kocks, 2006
CAT	5.695 units	2 units	Kon, 2014
CRQ	3.74 units	10 units	Cazzola, 2015
SF-12	3.825 units	3-3.5 units	Jones, 2012

Abbreviations: CAT, COPD Assessment Test; CCQ, Clinical COPD Questionnaire; CRQ, Chronic Respiratory Disease Questionnaire; MCID, minimal clinically important difference; SF, short form; SGRQ, St George's Respiratory Questionnaire

Jones, P. W. St. George’s respiratory questionnaire: MCID. in *COPD: Journal of Chronic Obstructive Pulmonary Disease* **2**, 75–79 (2005).

Kocks, J. W. H. *et al.* Health status measurement in COPD: The minimal clinically important difference of the clinical COPD questionnaire. *Respir. Res.* **7**, (2006).

Kon, S. S. C. *et al.* Minimum clinically important difference for the COPD Assessment Test: A prospective analysis. *Lancet Respir. Med.* **2**, 195–203 (2014).

Cazzola, M. *et al.* A review of the most common patient-reported outcomes in COPD – revisiting current knowledge and estimating future challenges. *International Journal of COPD* **10**, 725–738 (2015).

Jones, P. W. *et al.* Patient-centred assessment of COPD in primary care: Experience from a cross-sectional study of health-related quality of life in Europe. *Prim. Care Respir. J.* **21**, 329–336 (2012).

Supplementary Table 3

Author (Year)<br>Sample Size	Form of dyspnoea assessment reported	Group allocation	
		Intervention	*Control*
Chau (2012)<br>Intervention N=22<br>Control N=18	CRQ (Dyspnoea), mean (SD); p = NS	B: 4.27 (1.23)<br>F: 3.97 (1.17)	B: 4.20 (0.83)<br>F: 4.45 (0.96)
Nguyen (2013)<br>Intervention N=43<br>Control N=41	CRQ (Dyspnoea), mean (SD); p = NS	B: 23.8 (5.69)<br>F: 26 (8.36)	B: 23.3 (5.72)<br>F: 24 (7.19)
Tabak (2014 - B)<br>Intervention N=14<br>Control N=16	MRC, mean (SD); p = NS	B: 2.0 (0.9)<br>F: 1.7 (0.7)	B: 2.3 (1.4)<br>F: 2.1 (0.9)
Vorrink (2016)<br>Intervention N=84<br>Control N=73	CRQ (Dyspnoea), median (IQR); p = NS	B: 4.8 (1.3)<br>C: -0.17 (-0.44 to 0.09)	B: 4.8 (1.3)<br>C: -0.08 (-0.3 to 0.14)

Abbreviations: CRQ, Chronic Respiratory Disease Questionnaire; IQR, interquartile range; NS, non-significant; SD, standard deviation

Supplementary Table 4

Author (Year)<br>Sample Size	Form of fatigue assessment reported	Group allocation	
		Intervention	*Control*
Chau (2012)<br>Intervention N=22<br>Control N=18	CRQ (Fatigue), mean (SD); p = NS	B: 4.09 (1.26)<br>F: 4.11 (1.25)	B: 4.40 (0.99)<br>F: 4.79 (1.07)
Tabak (2014 - A)<br>Intervention N=12<br>Control N=12	MFI, mean (SD); p = NS	B: 12.3 (4.85)<br>F: 10.4 (4.5)	B:15.0 (4.85)<br>F: 13.8 (5.2)
Tabak (2014 - B)<br>Intervention N=14<br>Control N=16	MFI, mean (SD); p = NS	B: 12.5 (4.0)<br>F: 11.6 (2.7)	B: 11.6 (4.4)<br>F: 11.3 (2.6)
Vorrink (2016)<br>Intervention N=84<br>Control N=73	CRQ (Fatigue), mean (SD) or median (IQR); p = NS	B: 4.3 (1.1)<br>C: -0.14 (-0.35 to 0.07)	B: 4.2 (1.2)<br>C: -0.12 (-0.37 to 0.13)
Orme (2018)<br>Intervention N=12<br>Control N=11	FACIT, mean (SD); p = NS	B: 25.9 (11.1)<br>F: 25.9 (9.2)	B: 32.6 (9.1)<br>F: 34.0 (15.9)

Abbreviations: CRQ, Chronic Respiratory Disease Questionnaire; FACIT, Functional Assessment of Chronic Illness Therapy; IQR, interquartile range; MFI, Multidimensional fatigue inventory; NS, non-significant; SD, standard deviation

Supplementary Table 5

Author (Year)<br>Sample Size	Physical activity measurement	Group allocation	
		Intervention	*Control*
Tabak (2014 - A)<br>Intervention N=12<br>Control N=12	IMA, mean (SD) p = NS	B: 536.3 (147.6)<br>F: 511.0 (152.8)	B: 360.5 (154.8)<br>F: 335.2 (160.4)
Tabak (2014 - B)<br>Intervention N=14<br>Control N=16	Steps per day, mean (SD); p = NS	B: 5766 (3860)<br>F: 5603 (3856)	B: 5256 (3460)<br>F: 4617 (3460)
van der Weegen (2015)<br>Intervention N=65<br>Control N=68	Minutes per day, mean (SD); p<0.01	B: 39.29 (18.1)<br>F: 48.82 (23.8)	B: 44.13 (20.3)<br>F: 42.40 (18.9)
Vorrink (2016)<br>Intervention N=84<br>Control N=73	Steps per weekday, mean (SD); p = NS	B: 5824 (3418)<br>C: -1225 (-1712 to -730)	B: 5717 (2870)<br>C: -1148 (-1651 to -644)
Demeyer (2017)<br>Intervention N=171<br>Control N=172	Steps per day, mean (SD); p = NS	B: 4634 (2697)<br>C: +870 (2819)	B: 5120 (2932)<br>C: -678 (-1870)

Abbreviations: B, baseline; C, change; F, follow up; IMD, accelerometer counts per minute; SD, standard deviation

Supplementary Table 6

Author (Year)<br>Sample Size	Form of self-efficacy reported	Group allocation	
		Intervention	*Control*
Nguyen (2013)<br>Intervention N=43<br>Control N=41	Dyspnoea Management, mean (SD); p = NS	B: 4.93 (2.91)<br>F: 6.99 (3.08)	B: 5.74 (2.92)<br>F: 6.35 (3.14)
Pinnock (2013)<br>Intervention N=8<br>Control N=8	SECD6, mean (SD); p = NS	B: 5.0 (2.2)<br>F: 5.0 (2.2)	B: 5.2 (2.3)<br>F: 5.3 (2.5)
van der Weegen (2015)<br>Intervention N=65<br>Control N=68	General self-efficacy scale, mean (SD); p = NS	B: 3.2 (0.5)<br>F: 3.2 (0.5)	B: 3.1 (0.5)<br>F: 3.2 (0.4)
	Exercise self-efficacy, mean (SD); p = NS	B: 55.4 (17.0)<br>F: 52.1 (16.1)	B: 54.0 (19.2)<br>F: 56.5 (19.2)
Orme (2018)<br>Intervention N=12<br>Control N=11	FES-I, mean (SD); p = NS	B: 25.5 (9.5)<br>F: 34.3 (3.3)	B: 23.3 (10.5)<br>F: 26.3 (13.3)

Abbreviations: B, baseline; F, follow up; FESI, Falls Self-efficacy Scale-International; SECD6, Self-efficacy for Managing Chronic Disease ; NS, non-significant
